# Supplementary material for: In-depth investigations of size and occupancies in cobalt ferrite nanoparticles by joint Rietveld refinements of X-ray and neutron powder diffraction data
Source: J Appl Crystallogr. 2022 Oct 1;55(Pt 5):1336–50. doi: 10.1107/S1600576722008123 (PMC9533760; doi:10.1107/S1600576722008123)
Supplement: Supplementary file 1 [file j-55-01336-sup1.pdf]

JOURNAL OF APPLIED CRYSTALLOGRAPHY

Volume 55 (2022)

Supporting information for article:

In depth investigations of size and occupancies in cobalt ferrite nanoparticles by joint Rietveld refinements of X-ray and neutron powder diffraction data

Killian Henry, Jakob Ahlburg, Henrik Andersen, Cecilia Granados-Miralles, Marian Stingaciu, Matilde Saura-Múzquiz and Mogens Christensen

TABLE OF CONTENTS

Refinement details--2

Lorentzian isotropic size parameter, Y --2

Rietveld refinement summary ---2

Apparent crystallite size (ACS): ---3

Net intrinsic magnetisation ($M^{Neutron}$)--3

Comparative study on the effect of the Co:Fe magnetic moment ratio------------------------3

Part 1) Reliable extraction of Fe/Co occupancies in $CoFe_2O_4$ ----------------------------6

1.a) Pattern weighting influence --6

1.b) Combining different patterns --9

Part 2) Reproducibility study --- 10

Part 3) Effect of different synthesis approaches-- 15

Impact on the refinement of the anisotropic displacement parameters (ADP) for high Q-coverage neutron data. --- 15

3.a) Different hydrothermal reactors --- 18

3.b) Different cobalt salts -- 19

References --- 20

Refinement details

Lorentzian isotropic size parameter, Y

Lorentzian isotropic size parameter, Y, was constrained between patterns taking into account the differences in broadening as function of scattering angle due to the use of different wavelengths. This is illustrated in Table S1, where pattern #1 refers to Cu $K\alpha_1$, pattern #2 to Co $K\alpha_1$ and the last by DMC neutron source. The wavelength of the first pattern was chosen as default. The wavelength ratio between a pattern (#2 or #3) and the default pattern (#1) was used to describe the code to apply in the *FullProf Suite* software. This leads to $\lambda_{Co}/\lambda_{Cu}$= **1.161** and $\lambda_{DMC}/\lambda_{Cu}$= **1.596** for Co and DMC patterns, respectively. Furthermore, the value for the Lorentzian isotropic size parameter for the two last patterns has to be calculated using the wavelength ratio too. Hence, equations (S 1) and (S 2) leads to Y value of the two last patterns.

$$Y_{\mathrm{Co}} = Y_{\mathrm{Cu}} * \frac{\lambda_{\mathrm{Co}}}{\lambda_{\mathrm{Cu}}} = 0.424557 * 1.161 = \mathbf{0.492908} \qquad \text{(S 1)}$$

$$Y_{\mathrm{DMC}} = Y_{\mathrm{Cu}} * \frac{\lambda_{\mathrm{DMC}}}{\lambda_{\mathrm{Cu}}} = 0.424557 * 1.596 = \mathbf{0.677593} \qquad \text{(S 2)}$$

Table S1: Description of the Lorentzian isotropic size parameter Y and the constrained specific values for Co and DMC patterns. Bold numbers are calculated numbers.

	Cu	Co	DMC
λ	1.540593	1.788920	2.459525
Y	0.424557	**0.492908**	**0.677593**
Code for Y	1.000	**1.161**	**1.596**

Rietveld refinement summary

Table S2: Refinement details of the $CoFe_2O_4$ atomic- and magnetic structure used for the modelling in the *FullProf suite* software. Space group: *Fd*-3*m* (227). ***a***, ***b***, ***c***, ***d***, ***e***, and ***f*** are the FullProf code used for the refinement. The thermal vibrations were described by two different refinable B_{iso} in study **3)** and **4)**. Site occupancy of *Td* and *Oh* sites were refined separately, using the codes ***d*** and ***e***. In the case of study **1)** and **2)** thermal vibrations were described by B_{ov}, with the site occupancy of both sites refined jointly, which is represented by ***d*** and ***-d*** here. Occupancy for *Td* and *Oh* sites were calculated with respect the Co:Fe ratio of 1:2.

Atom	Atomic position			Thermal vibrations	Site occupancy	R_x
	x	y	z			
O^{2-}	x(O)<br>***a***	x(O)<br>***a***	x(O)<br>***a***	B_{iso}(O)<br>***b***	0.16667<br>/	/
Co^{2+} (*Td*)	3/8	3/8	3/8	B_{iso}(Fe/Co)<br>***c***	0.04166<br>***d***	-2.4<br>***0.6 f***
Fe^{3+} (*Td*)	3/8	3/8	3/8	B_{iso}(Fe/Co)<br>***c***	0.00001<br>***-d***	-4<br>***f***
Co^{2+} (*Oh*)	0	0	0	B_{iso}(Fe/Co)<br>***c***	0.00001<br>***e (-d)***	2.4<br>***-0.6 f***
Fe^{3+} (*Oh*)	0	0	0	B_{iso}(Fe/Co)<br>***c***	0.08332<br>***-e (d)***	4<br>***-f***

Apparent crystallite size (ACS):[1]

The ACS is used in the *FullProf Suite* software,[2,3] and is described as:

$$< D_{\mathbf{H}} > = \frac{\lambda}{\beta_{\mathbf{H}}\, cos\, \theta_{\mathbf{H}}} \quad \text{(S 3)}$$

Where $< D_{\mathbf{H}} >$ is the volume-weighted average domain size in the direction of the scattering vector, λ is the wavelength of the X-ray source, $\beta_{\mathbf{H}}$ is the integral breadth of the $\mathbf{H}^{th}$ reflection and $\theta_{\mathbf{H}}$ the Bragg angle of the $\mathbf{H}^{th}$ reflection.

Net intrinsic magnetisation ($M^{Neutron}$)

To determine the net intrinsic magnetisation ($M_{sat}^{Neutron}$), the atomic fraction of each atoms in the inverse spinel structure, $(Co^{2+}_{1-x}\, Fe^{3+}_{x})^{tet}\, [Co^{2+}_{x}\, Fe^{3+}_{2-x}]^{oct}\, O_4$, and the refined magnetic moment dipole moment R_x of both tetrahedral (*Td*) and octahedral (*Oh*) sites were considered as a function of the the formula unit (*f.u.*) $CoFe_2O_4$. We used the following equations:

$$m_{f.u.}(\mu_B/f.u.) = \left[(1-x)R_x^{\mathrm{Co}(Td)} + xR_x^{\mathrm{Fe}(Td)} + xR_x^{\mathrm{Co}(Oh)} + (2-x)R_x^{\mathrm{Fe}(Oh)}\right] \quad \text{(S 4)}$$

$$M_{sat}^{Neutron}(\mathrm{A.m^2/kg}) = \frac{\sum m}{\mathrm{mass}} = \frac{m(\mu_B/f.u.) * Na(f.u./\mathrm{mol}) * \mu_B(\mathrm{A.m^2})}{\mathrm{M}_{f.u.}(\mathrm{g/mol}) * 10^{-3}} \quad \text{(S 5)}$$

with $m_{f.u.}$ the formula unit magnetic moment, $M_{f.u.}$ the molecular mass of the formal unit (234.625 g/mol), N_A the Avogadro constant with $N_A = 6.022.10^{23}$ mol^{-1} and $\mu_B = 0.927.10^{-23}$ A.m^2.

Comparative study on the effect of the Co:Fe magnetic moment ratio

When refining the magnetic structure of $CoFe_2O_4$ an important question need to be asked: should we take into account the Co orbital contribution (μ_{S+L}(Co)) to the magnetic moment of Co and Fe? To answer that question we have investigated the effect of the Co:Fe magnetic moment ratio. Two extrem cases were considered: **1)** the cobalt orbital moment is supposed to be quenched, therefore only the number of unpaired electrons is used to described the magnetic moment of Co and Fe, which is 3 and 5, respectively. **2)** The orbital contribution of cobalt is considered, leading to Fe^{3+} having a magnetic moment of 5.9 (μ_S(Fe)), and Co^{2+} of 5.2 (μ_{S+L}(Co)).[4] A third refinement model was also investigated, where the constrainement of *Td* and *Oh* was raised, allowing both sites to be refined independently. The data used for this study were those from AC240 sample. The result of this investigation is summarized in Table S3 below.

Table S3: Comparison of the effect of different Co:Fe magnetic moment ratio. The ratio used is indicated between ( ). For the two first models, the magnetic moment *Td* and *Oh* sites are refined to be equals and opposite (*Td* have negatives values, while *Oh* positives). In the last model, AC240_Rx_Td-Oh, the constrainement is released and both sites are refined independently.

	AC240(3:5) PUS / Cu	AC240_Rx(5.2:5.9) PUS / Cu	AC240_Rx_Td-Oh PUS / Cu
Unit Cell (Å)	8.3925(1)	8.3925(1)	8.3925(1)
Cryst. Size (nm)	15.1(8)	15.1(8)	15.1(8)
Cryst. Size (nm) [25]		15.3(1)	
x(O)	0.2421(1)	0.2421(1)	0.2421(1)
B_{iso}(O) (Å²)	0.77(3)	0.76(3)	0.80(3)
B_{iso}(Fe/Co) (Å²)	1.09(2)	1.09(2)	1.08(2)
B_{ov}^{calc} (Å²)	0.91(2)	0.90(2)	0.92(2)
Occ(Co^{2+})^{Td} (%)	30(1)	30(1)	31(1)
Occ(Fe^{3+})^{Td} (%)	70(2)	70(2)	69(2)
Occ(Co^{2+})^{Oh} (%)	39(1)	39(1)	40(1)
Occ(Fe^{3+})^{Oh} (%)	61(1)	61(1)	60(1)
(Co^{2+}_{1-x} Fe^{3+}_{x})^{Td}	($Co_{0.30(1)}Fe_{0.70(2)}$)	($Co_{0.30(1)}Fe_{0.70(2)}$)	($Co_{0.31(1)}Fe_{0.69(1)}$)
[Co^{2+}_{y} Fe^{3+}_{2-y}]^{Oh}	[$Co_{0.78(1)}Fe_{1.22(2)}$]	[$Co_{0.77(1)}Fe_{1.23(2)}$]	[$Co_{0.80(1)}Fe_{1.20(2)}$]
Co:Fe ratio	1.08(2):1.92(3)	1.07(2):1.93(3)	1.12(2):1.88(3)
R_x(Co^{2+})^{Td} (μ_B)	-2.25(1)	-2.97(1)	-3.24(5)
R_x(Fe^{3+})^{Td} (μ_B)	-3.75(2)	-3.38(2)	-3.68(6)
R_x(Co^{2+})^{Oh} (μ_B)	2.25(1)	2.97(1)	2.72(5)
R_x(Fe^{3+})^{Oh} (μ_B)	3.75(2)	3.38(2)	3.10(5)
m (μ_B/f.u.)	3.0(1)	3.2(1)	2.4(1)
$M^{Neutron}$ (Am²/kg)	72(3)	76(3)	56(3)
M_{sat}^{VSM} (Am²/kg)		68.58(2)	
R_{wp} (%)	10.1 / 11.2	10.2 / 11.2	10.0 / 11.2
χ^2	2.04 / 1.47	2.05 / 1.47	2.00 / 1.47
R_{Bragg} (%)	3.50/6.66	3.59 / 6.66	3.32 / 6.70
R_{mag} (%)	3.92 / -	4.01 / -	3.77 / -

The R_x ratio modification does not interfere with the refinement of the structural properties, since the unit cell, oxygen position, ADP and occupancies are exactly the same as the previous study above. Undoubtedly, changing the initial 3:5 (0.6) ratio to 5.2:5.9 (0.88) increase the atomic magnetic moment of Co, but for Fe it was lowered, compared to AC240. Therefore, *m* and $M^{Neutron}$ are increased for AC240_Rx(5.2:5.9), but still within the same uncertainty. On the other hand, when the atomic magnetic moment assigned to *Td* and

Oh sites are refined individually, drastic changes are observed. Generally, $R_x(Fe^{3+})$ is lowered compared to the initial model, while $R_x(Co^{2+})$ has increased. Nonetheless, $R_x(Fe^{3+})^{Td}$ is within the same uncertainty as the first model, which is not the case for iron in *Oh* sites. Therefore, the calculated *m* and M^{Neutron} were both been reduced by ~20% compared to AC240. Thus, refining both sites individually does lead to a proper description of the magnetic phase of CFO, since the calculated magnetic moment M^{Neutron} does not correspond to the measured value M_{sat}^{VSM}.

In the end, two extreme cases were tested to describe the magnetic phase of $CoFe_2O_4$: a model based on the number of unpaired electron of both cobalt and iron, and another where the orbital magnetic moment is included. Regarding the refinement, it cannot be conclude which model is better. However, by comparing with our experimental data, we could argue that our initial model is in good agreement with macroscopic magnetisation data. Thus, our approximation of the orbit moment being quenched and the value of the magnetic moments being proportional to the number of unpair electrons independent of temperature is an adequate approximation.

Part 1) Reliable extraction of Fe/Co occupancies in $CoFe_2O_4$

1.a) Pattern weighting influence

In the present work, the PXRD patterns collected with the Co and Cu sources contain the same 18 reflections while the neutron data from DMC is limited to only 6 reflections. Three different weighting schemes have been evaluated for the combined refinement of all three patterns; First, an 'equal weight (Ew)' scheme was applied with the three patterns: DMC/Co/Cu being weighted 0.33/0.33/0.33. Secondly, an 'information weighted (Iw)' scheme was employed based on the number of reflections in each pattern divided by the sum of all reflections $Iw_{pattern} = \Sigma_{peaks\ in\ one\ pattern}/\Sigma_{peaks\ in\ all\ patterns}$, yielding a 0.14/0.43/0.43 weighting of the DMC/Co/Cu datasets. Finally, an ‘arbitrary weight (Aw)’ scheme was chosen to favour the weight of the neutron data over the two PXRD sources, with a weighting equal to 0.5/0.25/0.25. The powder diffraction patterns refined using the Ew model are shown in the manuscript (Fig. 1), while Figure S1 display the two other wheighting schemes. Notably, it is not possible to visually distinguish the refinement models based on the three weighting schemes. Table S4**Error! Reference source not found.** contains the results from the Rietveld refinements using the three weighting schemes.

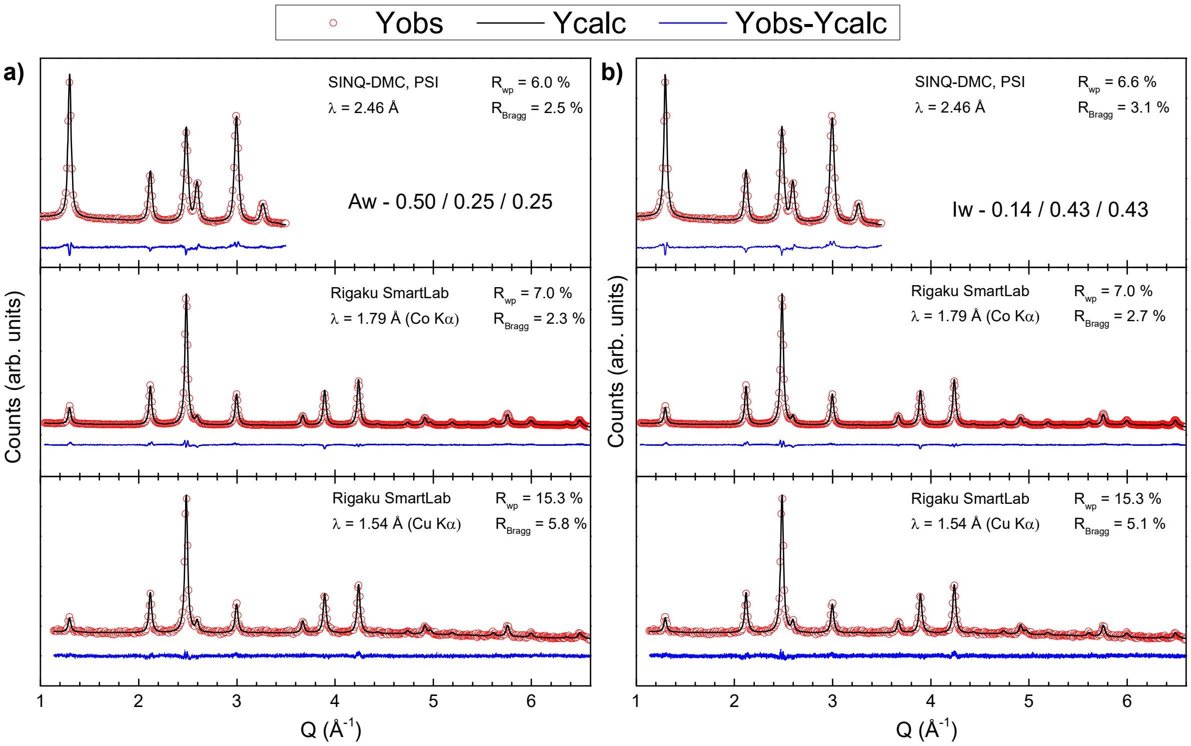

Figure S1: Combined Rietveld refinement of $CoFe_2O_4$ using **a)** 'arbitrary weighting Aw' and **b)** 'information weighting Iw' between diffraction patterns obtained using neutron, Co, and Cu as radiation sources. The data is shown by the red dots, the refined model by the black line and the residual by the blue line. Weighted profile and Bragg factors, respectively

R_{wp} and R_{Bragg}, are indicated for each diffraction pattern. For visualisation purpose, a frequency of 3 data points have been selected for NPD pattern, while the two PXRD patterns where drawn with a frequency of 15 points.

The three models give very similar results and/or are within the uncertainty of each other, indicating that the weight scheme does not hugely impact the refinements of the present data. It is observed that increasing the weight of the two PXRD sources slightly increases the obtained unit cell length, crystallite size and oxygen position, while the occupancy of Co^{2+} is lowered in the *Td* sites. The inversion degree is also higher with an increase of the PXRD data weight. The thermal vibrations, as well as the three magnetic parameters (R_x, *m* and *M*), are not affected by the weight modification as only the neutron data is providing information about these parameters.

Table S4: Comparison of three weighting schemes and their impact on the refined structural and magnetic parameters. The ‘arbitrary weight (Aw)’ is set to 0.50/0.25/0.25, while the 'equal weight (Ew)', model (0.33/0.33/0.33) refers to the default weighing system of the refinement software, and the 'information weighted (Iw)' (0.14/0.43/0.43) model is based on the number of reflections in each pattern. The saturation magnetisation extracted from a VSM measurement (M_{sat}^{VSM}) is tabulated along with the calculated formula unit magnetic moment ($m_{f.u.}$) of $CoFe_2O_4$ and the net intrinsic crystallographic magnetisation ($M^{Neutron}$). The numbers in parentheses represent the uncertainties of the FullProf Suite software, except for $M^{Neutron}$ where the uncertainties were calculated by the propagation of error. The number of reflections is written as ‘# reflections’ in the table.

	DMC / Co / Cu		
Weighted patterns	Aw<br>0.50 / 0.25 / 0.25	Ew<br>0.33 / 0.33 / 0.33	Iw<br>0.14 / 0.43 / 0.43
Unit Cell (Å)	8.3889(1)	8.3892(1)	8.3892(1)
Crystallite size (nm)	13.3(8)	13.2(8)	13.5(8)
x(O)	0.2425(1)	0.2425(1)	0.2427(1)
B_{ov} (Å^2)	1.07(1)	1.07(1)	1.07(1)
Occ(Co^{2+})^{Td} (%)	24(1)	24(1)	22(1)
Occ(Fe^{3+})^{Td} (%)	76(1)	76(1)	78(1)
Occ(Co^{2+})^{Oh} (%)	38(1)	38(1)	39(1)
Occ(Fe^{3+})^{Oh} (%)	62(1)	62(1)	61(1)
x	0.76(2)	0.76(2)	0.78(2)
R_x(Co^{2+})^{Oh} (μ_B)	2.33(1)	2.33(1)	2.34(2)
R_x(Fe^{3+})^{Oh} (μ_B)	3.89(2)	3.89(2)	3.89(3)
$m_{f.u.}$ (μ_B/f.u.)	3.1(1)	3.1(1)	3.0(1)
$M^{Neutron}$ (Am²/kg)	73(3)	73(2)	72(3)
M_{sat}^{VSM} (Am²/kg)		73.5(2)	
R_{wp} (%)	6.0 / 7.0 / 15.3	6.0 / 7.0 / 15.3	6.6 / 7.0 / 15.3
χ^2	4.8 / 4.8 / 0.9	4.7 / 4.8 / 1.0	5.6 / 4.8 / 0.9
R_{Bragg} (%)	2.5 / 2.3 / 5.8	2.5 / 2.8 / 5.1	3.1 / 2.7 / 5.1
R_{mag} (%)	0.96 / - / -	0.95 / - / -	1.32 / - / -
# reflections	6 / 18 / 18	6 / 18 / 18	6 / 18 / 18

Concerning the agreement factors (or *R*-factors), they are conventionally used as an indicator of the 'goodness of fit'. Thus, by lowering the agreement factors, a better fit should be obtained. Regarding **Error! Reference source not found.**, we clearly see that the *R*-factors of the Cu pattern are not influenced by the weight modification, contrary to Co and DMC patterns. However, the changes are small and does not exceed 1%. As shown here, simply using the agreement factors may not be an appropriate way to determine the optimal weighting scheme. In some cases, the obtained fit might get worse because the strengths of the included or more heavily weighted dataset highlights the shortcomings of the employed model. In that case, blindly trusting the *R*-factors will yield the best fit, but not necessarily an accurate result. Instead, it may be more intuitive to weight the patterns according to their individual strengths and the desired structural information. The Cu pattern carries the least information about the Co/Fe occupancies in the spinel structure. However, the Cu and Co patterns are rather important for the description of the lattice and microstructural parameters since they have the better peak and *Q*-range resolution. Consequently, to investigate the spinel inversion degree, as well as the structural properties, it is preferable to weight the neutron data higher in the refinement, but without neglected the PXRD data.

1.b) Combining different patterns

The diffraction pattern of the six remaining combination are display in Figure S2 below.

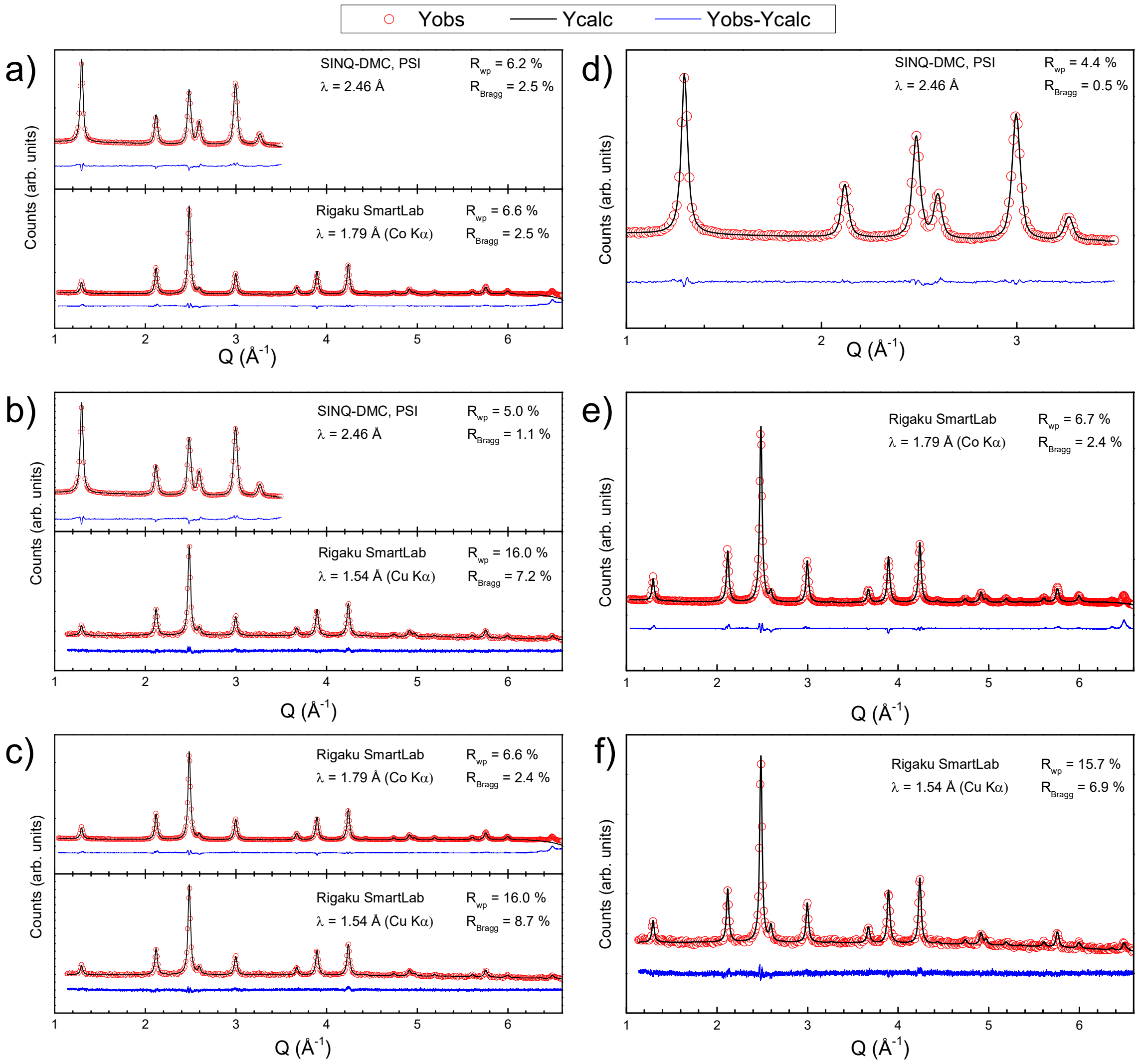

Figure S2: Diffraction pattern of the six remaining permutations with **a)** II (DMC/Co), **b)** III (DMC/Cu), **c)** IV (Co/Cu), **d)** V (DMC) **e)** VI (Co) and **f)** VII (Cu). The data is shown by the red dots, the refined model by the black line and the residual by the blue line. Weighted profile and Bragg factors, respectively R_{wp} and R_{Bragg}, are indicated for each diffraction pattern. A frequency of 3 data points have been selected for NPD pattern, while the two PXRD patterns where plotted with a frequency of 15 points.

Part 2) Reproducibility study

The PXRD and NPD data of samples A, B and C are plotted in Figures S3-S5, respectively. The additional peak observed around 3.1 Å^{-1} for C_3 was attributed to the (111) reflection of pure Ni, coming from a thermocouple used during the diffraction experiment. The peak present at 5.4 Å^{-1} in all PXRD data is attributed to the (222) reflection of the Al sample holder and was exclude from the refinement to improve the fit. The down sloping background starting at about Q = 5 Å^{-1} suggests a reduction in the probed sample volume, due to the beam fully penetrating the sample and hitting the aluminium sample holder below. This will cause the ADPs to be slightly overestimated.

In order to investigate the effect of the thermal vibration on the refinement of the atomic and microstructural parameters of $CoFe_2O_4$, the thermal vibration of sample C was fixed at 1.57 Å^2, corresponding to the refined value obtained in sample A. This constitute what we have named C_BovFIX sample. The results of the refinement of the data from sample C and sample C_BovFIX are gathered in Table S5.

The B_{ov} investigation has revealed that the thermal vibrations did not modify the refinement of C_BovFIX sample. Only minute deviations were recorded but considering the uncertainties, the deviation is too small to confirm that changing B_{ov} impacts the refinement.

Table S5: Data collection comparison of sample C. 1, 2 and 3 indicate the different measurements. Samples C_BisoFIX are the same samples as C, except that the B_{ov} was fixed at 1.57 Å², which correspond to the refined value obtained in samples A.

	C1<br>DMC / Co	C2<br>DMC / Co	C3<br>DMC / Co	C1_BovFIX<br>DMC / Co	C2_ BovFIX<br>DMC / Co	C3_ BovFIX<br>DMC / Co
Unit Cell (Å)	8.3919(1)	8.3919(1)	8.3919(1)	8.3920(1)	8.3920(1)	8.3920(1)
Cryst. Size (nm)	13.1(8)	13.1(8)	13.2(8)	13.1(8)	13.1(8)	13.1(8)
x(O)	0.2434(1)	0.2434(1)	0.2433(1)	0.2434(1)	0.2434(1)	0.2433(1)
B_{ov} (Å²)	1.44(1)	1.44(1)	1.44(1)	1.57(1)	1.57(1)	1.57(1)
Occ(Co^{2+})^{Td} (%)	19(1)	19(1)	19(1)	19(1)	19(1)	19(1)
Occ(Fe^{3+})^{Td} (%)	81(2)	81(2)	81(2)	81(2)	81(2)	81(2)
Occ(Co^{2+})^{Oh} (%)	40(1)	40(1)	40(1)	40(1)	40(1)	40(1)
Occ(Fe^{3+})^{Oh} (%)	60(1)	60(1)	60(1)	60(1)	60(1)	60(1)
x	0.81(2)	0.81(2)	0.81(3)	0.81(3)	0.81(2)	0.81(2)
R_x(Co^{2+})^{Oh} (μ_B)	2.70(6)	2.70(6)	2.56(5)	2.69(6)	2.69(6)	2.55(5)
R_x(Fe^{3+})^{Oh} (μ_B)	4.50(10)	4.51(10)	4.27(8)	4.48(11)	4.50(10)	4.25(8)
$m_{f.u.}$ (μ_B/f.u.)	3.4(2)	3.4(2)	3.2(2)	3.4(2)	3.4(2)	3.2(2)
$M^{Neutron}$ (Am²/kg)	81(5)	81(5)	77(5)	80(5)	81(5)	76(5)
M_{sat}^{VSM} (Am²/kg)	73.9(1)					
R_{wp} (%)	20.2 / 6.1	20.1 / 6.0	21.4 / 6.1	20.7 / 6.12	20.6 / 6.1	21.9 / 6.1
χ^2 (%)	1.1 / 4.2	1.2/ 4.2	2.5 / 4.2	1.1 / 4.25	1.2 / 4.3	2.5 / 4.3
R_{Bragg} (%)	1.5 / 2	3.8 / 2	5.5 / 2	1.71 / 2.21	3.7 / 2.2	5.5 / 2.2
R_{mag} (%)	3.3 / -	5.0 / -	6.3 / -	3.3 / -	4.9 / -	6.1 / -
# reflections	6 / 16	6 / 16	6 / 16	6 / 16	6 / 16	6 / 16

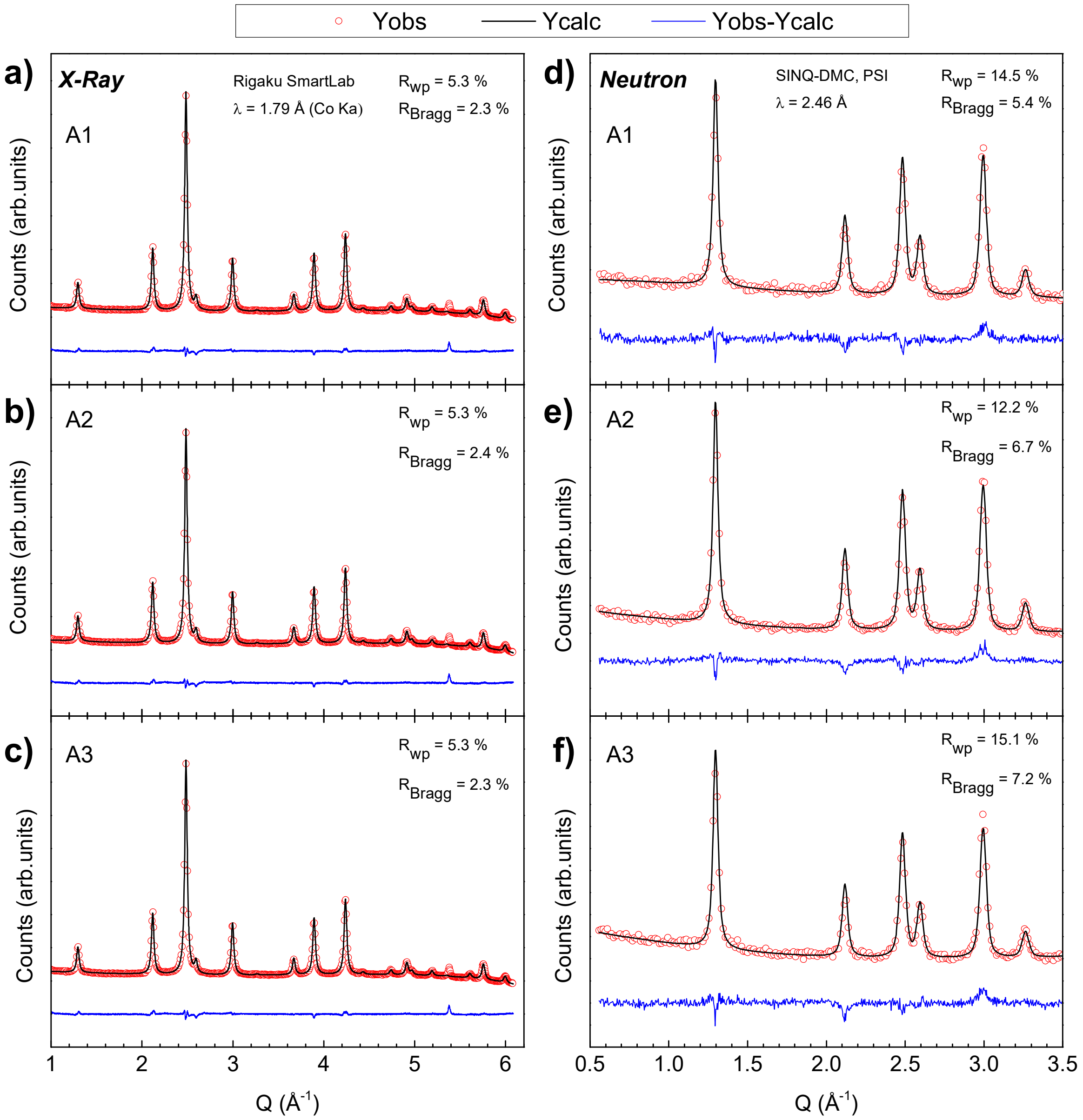

Figure S3: **a), b)** and **c)** are the PXRD data of samples A1, A2 and A3, respectively, while **d), e)** and **f)** represent the NPD data. The experimental data is shown by the red dots, the refined model by the black line and the residual by the blue line. The weighted profile and Bragg *R*-factors are indicated for each diffraction pattern. Frequencies of 3 and 15 data points were selected to plot the NPD and PXRD patterns, respectively.

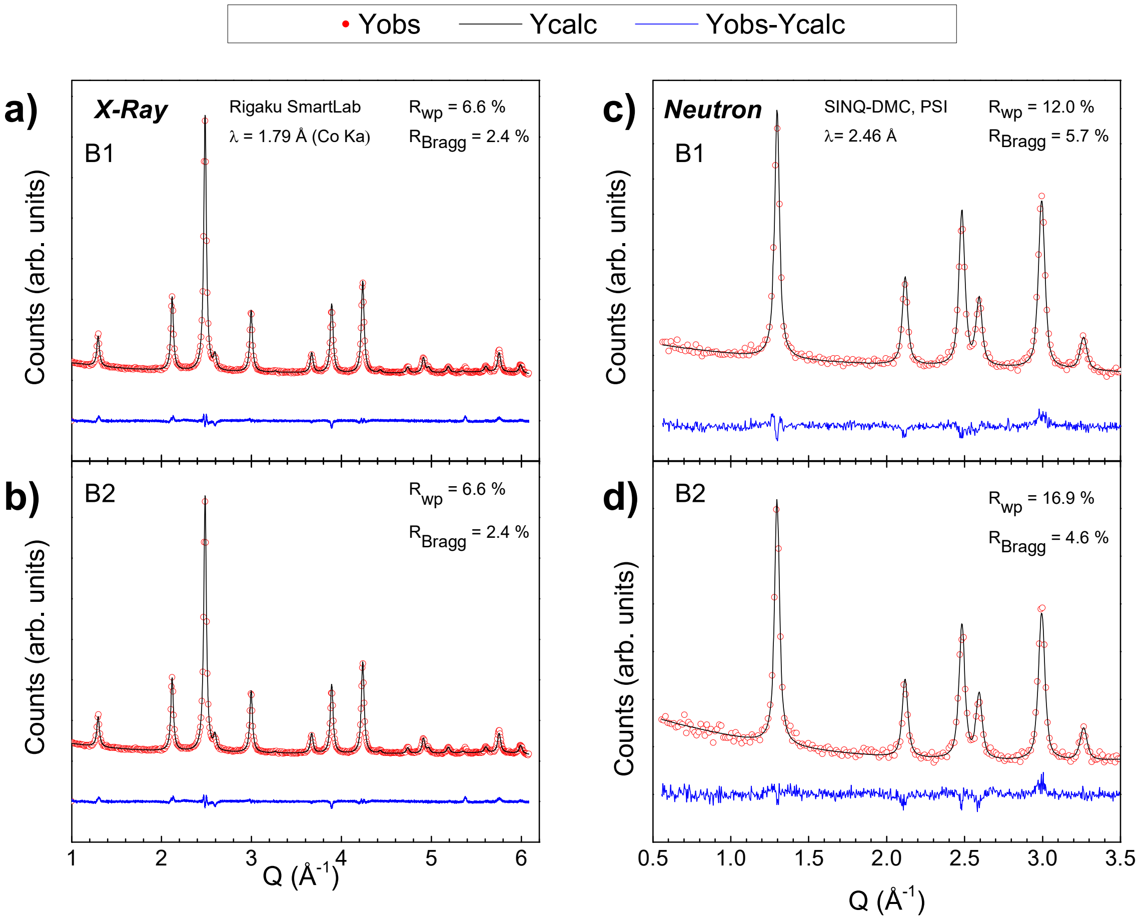

Figure S4: PXRD data of samples B1 (**a)**) and B2 (**b)**) and their corresponding NPD data (**c)** and **d)**, repectively). The data is shown by the red dots, the refined model by the black line and the residual by the blue line. For visualisation purpose, frequencies of 3 and 15 data points have been selected for NPD and PXRD patterns, respectively.

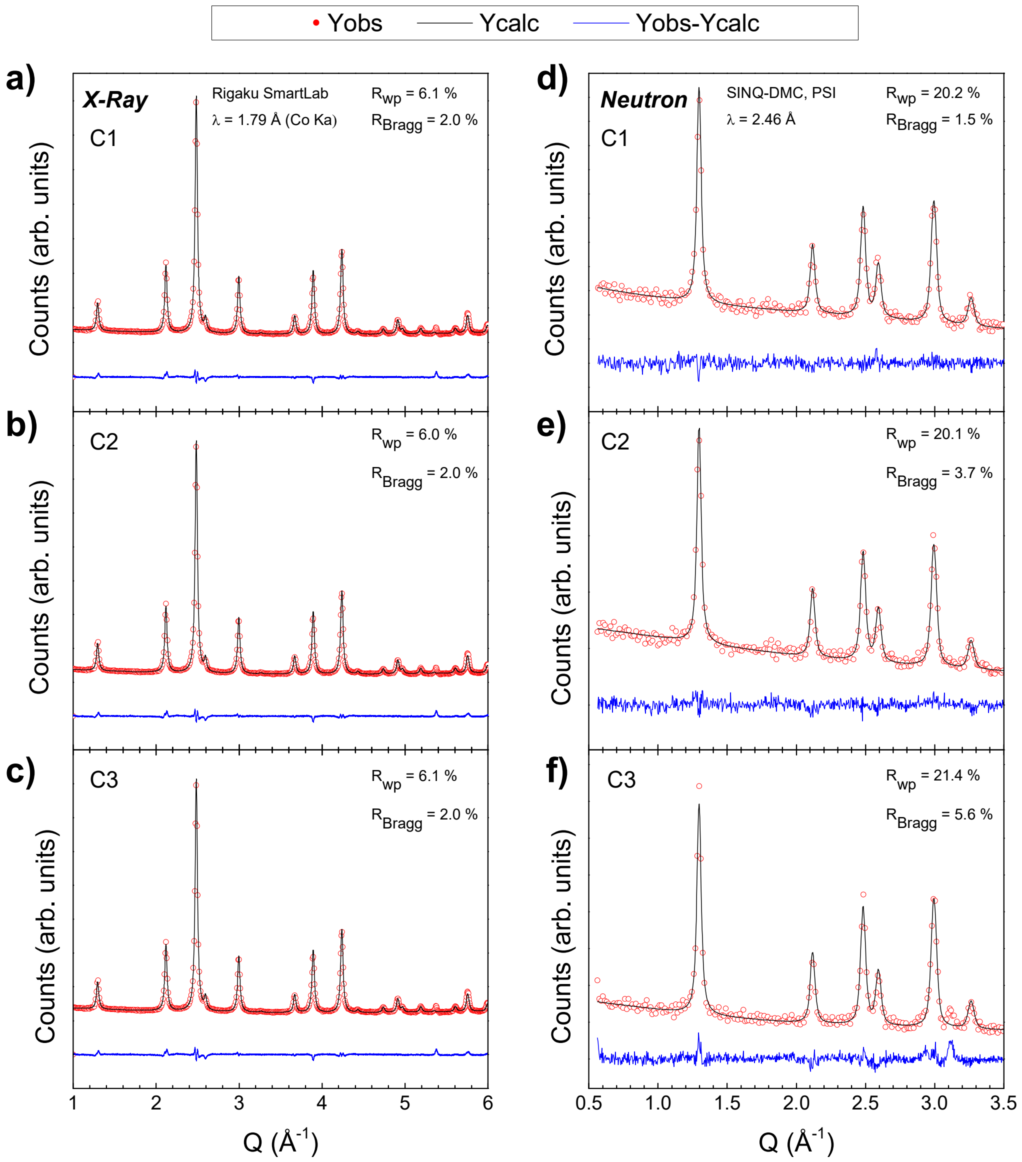

Figure S5: **a), b)** and **c)** are the XRPD data of samples C1, C2 and C3, respectively. Corresponding NPD data are **d), e)** and **f)** for samples C1, C2 and C3. The data is shown by the red dots, the refined model by the black line and the residual by the blue line. Only a frequencies of 3 and 15 data points were selected to draw the NPD and PXRD patterns, respectively.

Part 3) Effect of different synthesis approaches

Impact on the refinement of the anisotropic displacement parameters for high *Q*-coverage neutron data.

By using higher *Q*-coverage neutron data, one should expect to obtain more information, since more diffraction peaks are available. This addition of information on the neutron data could possibly bring a degree of freedom on the refinement of $CoFe_2O_4$. For this study, we have investigated the impact of two different refinements of the anisotropic displacement parameters. The first model, the one use in the main text, is based on B_{ov}, where all atoms are describe by a single parameter. Then the second model use two distinct isotropic ADPs, one for oxygen atoms, B_{iso}(O), and another for the metal ions, B_{iso}(Fe/Co). Table S6 below summarize the results obtained for both models on samples FR220, FR320, SR240 and AC240. As expected, the major variation observed are for *x*(O), ADP, occupancy and the magnetic parameters.

Anisotropic Displacement Parameter (B_{ov}): When addind a degree of freedom by using two B_{iso}, a decrease of the ADP is seen (B_{ov}^{calc}) and is equal for all samples, expect FR220 which is equal to ~1.2 Å². Comparing with the first study presented in the main text (**Part 1.b) Combining different patterns**) B_{ov} for FR320, SR240 and AC240 match very well with the result of model III (DMC/Cu; 0.89(4) Å²). Regarding this comparison, it seems that using a refinement combining NPD data with PXRD data obtained from Cu source yields to a similar description of the ADP, independent of the *Q*-coverage of the NPD data. In fact, this effect could be explained by the fact that the ADP are strongly correlated to background fitting, especially at high *Q* coverage. Thus, the fluorescence induced by Cu source may explain the refinement differences. Contrary to theory and what is usually observed in the literature for $CoFe_2O_4$,[5–8] B_{iso}(O) have a smaller amplitude than B_{iso}(Fe/Co), probably due to vacancies on the *Oh* and *Td* sites. As stated in **Part 1b)**, the occupancies from PXRD data are strongly correlated with the ADP. Considering the overall parameter (B_{ov}^{calc}), the refined values are within the range expected for inorganic compounds (~0.5 to ~3 Å²),[9] and also in adequation with those found in the above cited references, *i.e.* 0.57 – 1.05 Å².

Site occupancy: All samples have the same tetrahedral site occupancy, with Fe^{3+} occupying 70%, which is close to a random occupancy (x = 2/3) for a stoichiometry of Fe:Co = 2:1.[10,11] Different refined occupancies were obtained for the *Oh* site across the four samples. However, the refined parameters of FR220 substantially differs from the other samples, since sample FR220 have the highest amount of Co^{2+}, highest thermal vibration and smallest unit cell. The presence of vacancies in the structure could actually explain these results, as a

vacancy structure would also be expected to have a smaller unit cell.[12] Notably, spinel ferrite nanoparticles have previously been reported to have vacancies within the *Oh* site. As the *Oh* site is fixed to be fully occupied, the refinement can only reduce the site scattering power by introducing more Co^{2+} on the *Oh* site, or increase the ADP. Vacancies would also reduce the magnetic moment.

Since the ADPs are linked to the occupancy, it is therefore logical to see variation in the magnetic properties. By using two B_{iso}, the occupancy of *Td* sites have a trend to be equal to a 30:70 ratio, while for *Oh* sites the occupancy is between 30:70 and 40:60, depending on the sample. Undoubtly, the fraction of Fe in the refinement is increased by using the second model. The resulting effect of these variations, compared to the B_{ov} model, is higher atomic magnetic moment for both metals, leading to a $M^{Neutron}$ slightly higher. Comparing $M^{Neutron}$ to the VSM measurements reveal that the second model deviates from the experimental data, having the consequence that B_{ov} model gives a $M^{Neutron}$ value closer to the saturation magnetisation M_{sat}^{VSM}. Nonetheless, both parameters are equal regarding the uncertainty. For the *R*-factors, no significant changes are observed, except for R_{mag} which is lower for the first model.

To summarize, refining the ADP individually or by an overall parameter does not change drastically the refinement of the CFO sample. The only modification noticed were about the occupancy and the magnetic moment. In the end, using either two distinct B_{iso} or an overall B_{ov} parameter to describe the ADPs for high *Q*-coverage neutron data both appear appropriated for refining the $CoFe_2O_4$ data. It is not diretly evident, which of these two models gives a better description of the $CoFe_2O_4$ phase. Indeed, even if the second model is in better adequation with the VSM data, both models are equals with the error.

Table S6: Comparison on the effect of anisotropic displacement parameters on the structural and magnetic parameters of FR220 and FR320 samples. First model (sample name) used B_{ov}, while the second model is described by two distinct isotropic displacement parameters, one for oxygen, and another for the two metals.

	FR220_Bov<br>PUS / Cu	FR220_Biso<br>PUS / Cu	FR320_Bov<br>PUS / Cu	FR320_Biso<br>PUS / Cu
Unit Cell (Å)	8.3532(4)	8.3532(4)	8.3785(2)	8.3785(2)
Cryst. Size (nm)	5.2(8)	5.2(8)	10.9(8)	10.9(8)
Cryst. Size (nm)[25]	8.2(1)		10.6(1)	
x(O)	0.2417(2)	0.2414(2)	0.2413(1)	0.2409(1)
B_{iso}(O) (Å²)	-	0.94(5)	-	0.62(4)
B_{iso}(Fe/Co) (Å²)	-	1.52(3)	-	1.38(2)
B_{ov}^{calc} (Å²)	1.40(2)	1.19(3)	1.20(2)	0.94(3)
Occ$(Co^{2+})^{Td}$ (%)	35(2)	29(2)	37(3)	29(1)
Occ$(Fe^{3+})^{Td}$ (%)	65(3)	71(5)	63(4)	71(3)
Occ$(Co^{2+})^{Oh}$ (%)	45(2)	41(2)	39(1)	32(1)
Occ$(Fe^{3+})^{Oh}$ (%)	55(3)	59(3)	61(1)	68(3)
$(Co^{2+}{}_{1-x}\,Fe^{3+}{}_{x})^{Td}$	$(Co_{0.35(2)}Fe_{0.65(3)})$	$(Co_{0.29(2)}Fe_{0.71(5)})$	$(Co_{0.37(3)}Fe_{0.63(4)})$	$(Co_{0.29(1)}Fe_{0.71(3)})$
$[Co^{2+}{}_{y}\,Fe^{3+}{}_{2-y}]^{Oh}$	$[Co_{0.90(2)}Fe_{1.10(3)}]$	$[Co_{0.82(4)}Fe_{1.18(6)}]$	$[Co_{0.78(1)}Fe_{1.22(2)}]$	$[Co_{0.64(3)}Fe_{1.36(6)}]$
Co:Fe ratio	1.26(5):1.74(6)	1.12(5):1.88(8)	1.16(3):1.84(5)	0.94(3):2.06(7)
$R_x(Co^{2+})^{Oh}$ (μ_B)	2.10(3)	2.16(4)	2.16(2)	2.24(2)
$R_x(Fe^{3+})^{Oh}$ (μ_B)	3.49(6)	3.59(6)	3.59(4)	3.74(4)
m (μ_B/f.u.)	2.7(2)	2.8(3)	3.0(2)	3.2(3)
$M^{Neutron}$ (Am²/kg)	65(6)	67(8)	72(4)	76(7)
M_{sat}^{VSM} (Am²/kg)	38.68(2)		66.33(2)	
R_{wp} (%)	22.6 / 14.5	22.1 / 14.4	16.5 / 14.5	16.2 / 14.3
χ^2	1.6 / 2.47	1.6 / 2.5	1.25 / 1.76	1.2 / 1.7
R_{Bragg} (%)	11.0 / 6.59	10.6 / 6.8	7.50 / 10.3	7.1 / 10
R_{mag} (%)	13.6 / -	16.3 / -	9.26	11.6 / -

Table S7: Comparison on the effect of anisotropic displacement parameters on the structural and magnetic parameters of SR240 and AC240 samples. First model (sample name) used B_{ov}, while the second model is described by two distinct isotropic displacement parameters, one for oxygen, and another for the two metals.

	SR240_Bov<br>PUS / Cu	SR240_Biso<br>PUS / Cu	AC240_Bov<br>PUS / Cu	AC240_Biso<br>PUS / Cu
Unit Cell (Å)	8.3866(2)	8.3866(2)	8.3925(1)	8.3925(1)
Cryst. Size (nm)	10.7(8)	10.7(8)	15.1(8)	15.1(8)
Cryst. Size (nm)[25]	11.6(1)		15.3(1)	
x(O)	0.2419(1)	0.2414(2)	0.2423(1)	0.2421(1)
B_{iso}(O) (Å²)	-	0.62(3)	-	0.77(3)
B_{iso}(Fe/Co) (Å²)	-	1.34(2)	-	1.09(2)
B_{ov}^{calc} (Å²)	1.14(2)	0.93(3)	1.00(1)	0.91(2)
Occ(Co^{2+})^{Td} (%)	38(1)	30(1)	33(1)	30(1)
Occ(Fe^{3+})^{Td} (%)	62(1)	70(2)	67(1)	70(2)
Occ(Co^{2+})^{Oh} (%)	41(1)	35(1)	42(1)	39(1)
Occ(Fe^{3+})^{Oh} (%)	59(1)	65(2)	58(1)	61(1)
$(Co^{2+}_{1-x}\,Fe^{3+}_{x})^{Td}$	$(Co_{0.38(1)}Fe_{0.62(2)})$	$(Co_{0.30(1)}Fe_{0.70(2)})$	$(Co_{0.33(1)}\,Fe_{0.67(1)})$	$(Co_{0.30(1)}Fe_{0.70(2)})$
$[Co^{2+}_{y}\,Fe^{3+}_{2-y}]^{Oh}$	$[Co_{0.81(1)}Fe_{1.19(1)}]$	$[Co_{0.70(2)}Fe_{1.30(3)}]$	$[Co_{0.84(1)}\,Fe_{1.16(1)}]$	$[Co_{0.78(1)}Fe_{1.22(2)}]$
Co:Fe ratio	1.19(1):1.81(2)	1.00(2):2.00(4)	1.17(1):1.83(2)	1.08(2):1.92(3)
R_x(Co^{2+})^{Oh} (μ_B)	2.15(1)	2.22(1)	2.22(1)	2.25(1)
R_x(Fe^{3+})^{Oh} (μ_B)	3.58(2)	3.70(2)	3.70(2)	3.75(2)
m (μ_B/f.u.)	3.0(1)	3.1(2)	3.0(1)	3.0(1)
$M^{Neutron}$ (Am²/kg)	70(2)	74(4)	70(2)	72(3)
M_{sat}^{VSM} (Am²/kg)	62.77(2)		68.58(2)	
R_{wp} (%)	12.4 / 11.5	12.1 / 11.1	10.30 / 11.20	10.10 / 11.20
χ^2	3.65 / 1.48	3.5 / 1.4	2.09 / 1.50	2.04 / 1.50
R_{Bragg} (%)	7.47 / 4.52	7.2 / 4.7	3.80 / 6.90	3.50 / 6.66
R_{mag} (%)	8.84	8.87 / -	4.56 / -	3.92 / -

3.b) Different cobalt salts

The powder diffraction patterns for the different Co-precursors are shown in Figure S6.

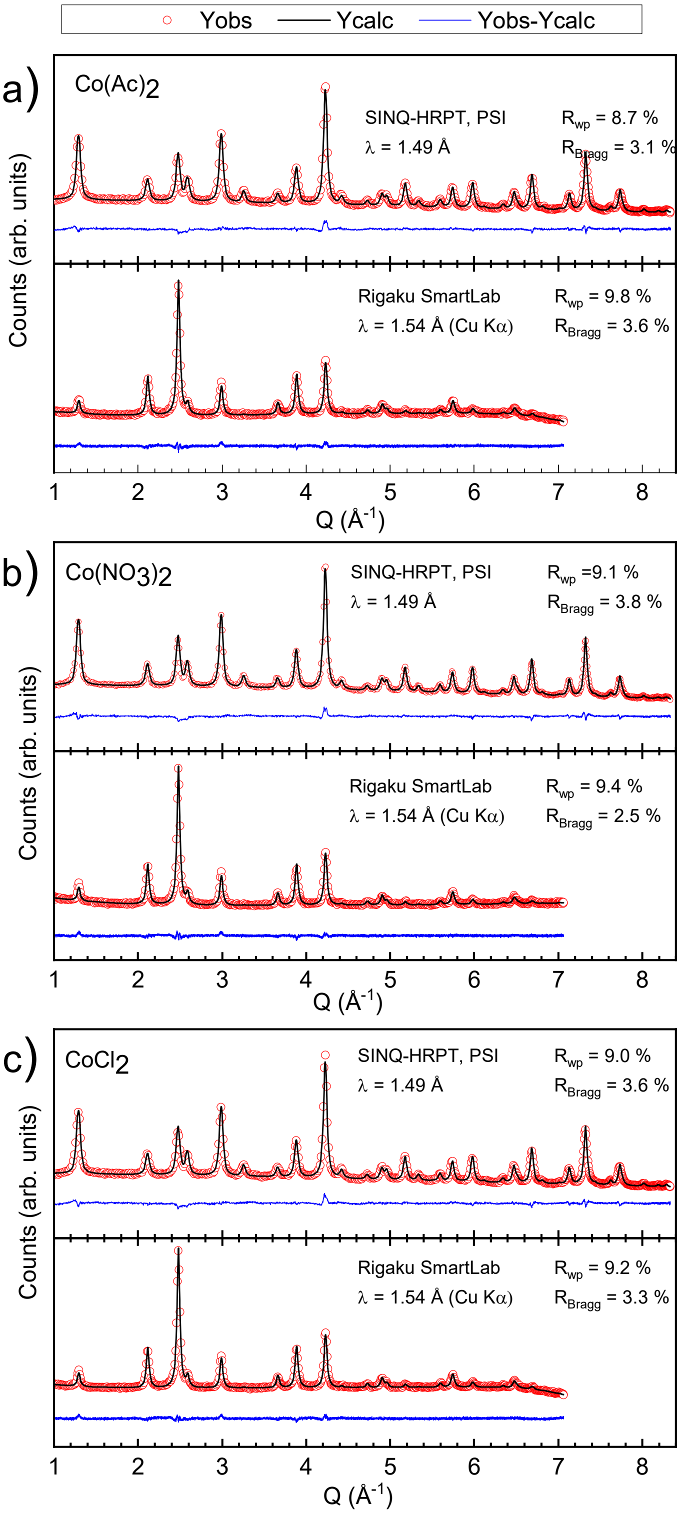

Figure S6: Diffraction patterns from $CoFe_2O_4$ synthesized using **a)** $Co(Ac)_2$, **b)** $Co(NO_3)_2$, **c)** $CoCl_2$. The data is shown by the red dots, the refined model by the black line and the residual by the blue line. Weighted profile and Bragg factors, respectively R_{wp} and R_{Bragg}, are indicated for each diffraction pattern. For visualisation purpose, frequencies of 4 and 15 data points have been selected for NPD and PXRD patterns, respectively.

References

1. Langford JI, Wilson AJC. Scherrer after sixty years: A survey and some new results in the determination of crystallite size. *J Appl Crystallogr*. 1978;11(2):102-113. doi:10.1107/S0021889878012844

2. Rodríguez-Carvajal J. Recent advances in magnetic structure determination by neutron powder diffraction. *Phys B Condens Matter*. 1993;192(1):55-69. doi:10.1016/0921-4526(93)90108-I

3. Rodríguez-Carvajal J. Introduction to the program FullProf. Published online 2003:24.

4. West AR. *Solid State Chemistry and Its Applications*. Second edition, student edition. Wiley; 2014.

5. Ferreira TAS, Waerenborgh JC, Mendonça MHRM, Nunes MR, Costa FM. Structural and morphological characterization of FeCo2O4 and CoFe2O4 spinels prepared by a coprecipitation method. *Solid State Sci*. 2003;5(2):383-392. doi:10.1016/S1293-2558(03)00011-6

6. Waseda Y, Shinoda K, Sugiyama K. Cation Distribution of ZnFe2O4 and CoFe2O4 Spinels From Anomalous X-Ray Scattering. *Z Für Naturforschung A*. 1995;50(12):1199-1204. doi:10.1515/zna-1995-1226

7. Shanmugavani A, Selvan RK, Layek S, Vasylechko L, Sanjeeviraja C. Influence of pH and fuels on the combustion synthesis, structural, morphological, electrical and magnetic properties of CoFe2O4 nanoparticles. *Mater Res Bull*. 2015;71:122-132. doi:10.1016/j.materresbull.2015.04.008

8. Tanaka M, Katsuya Y, Sakata O. Rietveld analysis using powder diffraction data with anomalous scattering effect obtained by focused beam flat sample method. *AIP Conf Proc*. 2016;1741(1):050019. doi:10.1063/1.4952939

9. Structure Factor. In: *Fundamentals of Powder Diffraction and Structural Characterization of Materials*. Springer, Boston, MA; 2009:203-238. Accessed September 9, 2020. https://link.springer.com/chapter/10.1007/978-0-387-09579-0_9

10. Sickafus KE, Wills JM, Grimes NW. Structure of Spinel. *J Am Ceram Soc*. 1999;82(12):3279-3292. doi:https://doi.org/10.1111/j.1151-2916.1999.tb02241.x

11. Sorescu M, Jubeck J, Knauss M, Perrin A, McHenry M. Effect of graphene on the mechanochemical activation of cobalt ferrite nanoparticles. *J Phys Chem Solids*. 2021;150:109866. doi:10.1016/j.jpcs.2020.109866

12. Ahlburg JV, Granados-Miralles C, Gjørup FH, Andersen HL, Christensen M. Exploring the direct synthesis of exchange-spring nanocomposites by reduction of $CoFe_2O_4$ spinel nanoparticles using *in situ* neutron diffraction. *Nanoscale*. 2020;12(17):9440-9451. doi:10.1039/D0NR00912A
